# SWAT-1: The effectiveness of a ‘site visit’ intervention on recruitment rates in a multi-centre randomised trial

**DOI:** 10.1186/s13063-015-0732-z

**Published:** 2015-05-10

**Authors:** Valerie Smith, Mike Clarke, Cecily Begley, Declan Devane

**Affiliations:** School of Nursing and Midwifery, Trinity College Dublin, D’Olier Street, Dublin 2, Ireland; Northern Ireland Network for Trials Methodology Research, Queen’s University Belfast, Grosvenor Road, Belfast, BT12 6BA UK; School of Nursing and Midwifery, National University of Ireland, Galway, Ireland

**Keywords:** Study Within A Trial, SWAT-1, multi-centre randomised trials

## Abstract

**Background:**

Recruitment rates in multi-centre randomised trials often fall below target recruitment rates, causing problems for study outcomes. The Studies Within A Trial (SWAT) Programme, established by the All-Ireland Hub for Trials Methodology Research in collaboration with the Medical Research Council Network of Hubs in the United Kingdom and others, is developing methods for evaluating aspects of trial methodology through the conduct of research within research. A recently published design for a SWAT-1 provides a protocol for evaluating the effect of a site visit by the principal investigator on recruitment in multi-centre trials.

**Methods:**

Using the SWAT-1 design, the effect of a site visit, with the sole purpose of discussing trial recruitment, on recruitment rates in a large multicentre trial in the Republic of Ireland was evaluated. A controlled before and after intervention comparison was used, where the date of the site visit provides the time point for the intervention, and for the comparison to control sites. Site A received the intervention. Site B and Site C acted as the controls. *Z*-scores for proportions were calculated to determine within site recruitment differences. Odds ratios and 95% confidence intervals were calculated to determine between site recruitment differences.

**Results:**

Recruitment rates were increased in Site A post-intervention (17% and 14% percentage point increases at 1 and 3 months, respectively). No differences in recruitment occurred in Site B or in Site C. Comparing between site differences, at 3 months post-intervention, a statistically significant difference was detected in favour of higher recruitment in Site A (34% versus 25%; odds ratio 1.57, 95% confidence interval 1.09 to 2.26).

**Conclusions:**

This is the first reported example of a study in the SWAT programme.. It provides evidence that a site visit, combined with a scheduled meeting, increases recruitment in a clinical trial. Using this example, other researchers might be encouraged to consider conducting a similar study, allowing the findings of future SWAT-1s to be compared and combined, so that higher level evidence on the effect of a site visit by the principal investigator can be obtained.

**The ADCAR trial:**

ISRCTN-96340041 (www.controlled-trials.com); date of registration: 25 March 2008.

## Background

Recruitment in large multi-centre randomised trials invariably presents a challenge for the research team. Actual recruitment rates often fall below those that were hoped for at the start of the trial [1-3]. Poor or slow recruitment causes problems for study outcomes, including a reduction in the statistical power of the study, delays in resolving the uncertainty that underpinned the need for the trial [1-4] and potential resource wastage. The clinical implication of an underpowered trial is the possibility that the study findings will conclude erroneously that there is no difference between the treatments under investigation when in reality there is a difference. This erroneous conclusion has clinical significance whereby people may be denied a potentially more effective treatment than that which was previously offered [1,2]. Poor or slow recruitment may also require extending the trial, which may lead to increased costs and implications for potential future funding by the funding agency [1].

Faced with poor or slow recruitment, trial researchers often undertake activities aimed strategically at improving recruitment. Examples of these include increased communication between the researchers and the study sites [5], incentives for trial recruiters or participants [6], modification to the training given to the recruiters [7] and modifications to the recruitment approach [8]. Responsive activities might be implemented as a single activity or as multiple activities at the same time. When the latter occurs, it is often difficult to disentangle the activities that may or may not improve recruitment rates in a trial.

### Studies Within A Trial (SWAT) Programme

Established by the All-Ireland Hub for Trials Methodology Research in collaboration with the Medical Research Council Network of Hubs in the United Kingdom and others, the SWAT (Studies Within A Trial) Programme is developing methods for evaluating the effects of different ways of designing, conducting, analysing and evaluating studies through the conduct of research within research [9]. The SWAT Programme is developing a series of short outlines for studies within trials that will be made available online for researchers to consider. The first in the series, SWAT-1, provides a protocol for evaluating the effects of site visits by the principal investigator on recruitment rates in multi-centre randomised trials [10]. Using SWAT-1 methodology, in this paper, we describe the effect of a site visit combined with a scheduled meeting, with the sole purpose of discussing recruitment, on recruitment rates in a multi-centre randomised trial (the ADCAR trial) in the Republic of Ireland.

### Aim

The aim of this study is to evaluate the effectiveness of a site visit combined with a scheduled meeting, with the sole purpose of discussing recruitment on recruitment rates to the ADCAR trial.

## Methods

### Context for the SWAT-1

The ADCAR trial is a multi-centre, three-site randomised trial that was conducted in the Republic of Ireland between May 2008 and April 2012. ADCAR evaluated the effectiveness of admission cardiotocography (ACTG) versus intermittent auscultation (IA) of the fetal heart rate (FHR) in low risk women on admission to the labour assessment room with signs of possible labour. Based on sample size estimates, a target study sample of 5,776 was required, and it was anticipated that this could be achieved within a 28-month recruitment time-frame. However, as the trial progressed, monthly overall recruitment rates averaged approximately one-third of target rates, with recruitment in some sites proportionately better than in others.

### Design

The study uses SWAT-1 methodology [10], with a controlled before and after intervention comparison where the date of the site visit provides the time point for the intervention and for the comparison to control sites. The study was retrospective in design whereby a decision was made to map the pre- and post-intervention recruitment rates to the site visit and scheduled meeting approximately eight months after the intervention was implemented. For quality in reporting our methods, we provide an assessment of the methodological quality of our study using the Cochrane Effective Practice and Organisation of Care (EPOC) Group risk of bias criteria (Table 1). Site A was chosen to receive the intervention because, although it had previously been achieving monthly recruitment of 30-40% of its initial target, this had fallen considerably in the months preceding the intervention to 25% or less of the target. Site B and Site C did not receive the intervention and acted as the controls.

**Table 1:** **Cochrane Effective Practice and Organisation of Care (EPOC) risk of bias criteria**

**Risk of bias criterion**	**Judgement**	**Reason for judgement**
Allocation sequence generation	High risk	Non-randomised method used
Allocation concealment	High risk	Controlled before and after study
Similarity in baseline outcome measurements	High risk	Although all sites had low recruitment prior to the intervention, this was imbalanced across the sites
Similarity in baseline characteristics	Low risk	Similar recruitment processes across sites
Incomplete outcome data addressed	Low risk	No missing data
Knowledge of the allocated intervention	Low risk	Objective outcomes, not affected by knowledge of the intervention
Protection against contamination	Low risk	Allocation was by study site
Free from selective reporting	Low risk	All pre-specified outcomes are reported
Free from other risks of bias	Unclear risk	Possible risk from confounders

### Description of the intervention

The intervention consisted of a site visit by the lead researcher, that is, the person responsible for the day-to-day management of the trial, combined with a scheduled meeting arranged with the sole purpose of discussing recruitment rates to the trial. The clinical manager at Site A was contacted with a request to facilitate the visit and meeting. This included advertisement of the meeting 2 weeks in advance, targeting senior midwifery and obstetric staff to attend the meeting and setting the meeting date and time to facilitate maximum staff attendance. The site visit took place on 22 June 2011. The meeting consisted of a 10-minute presentation inclusive of information on overall trial recruitment rates and site-specific recruitment trends followed by 20 minutes of discussion on challenges to recruitment and how these might be overcome. Table 2 provides additional details on the intervention meeting.

**Table 2:** **Detailed description of the intervention meeting**

**Detail**	**Description**
Date and Time	22 June 2011 at 9am
Attendance	18 clinical staff members directly involved in the recruitment process (that is, distributing information to potential participants, screening for eligibility and randomising those eligible and consenting). The staff mix was consultant obstetricians, obstetric registrar, senior house officers and senior midwives (midwifery managers and staff midwives with greater than 5 year’s clinical experience).
PowerPoint presentation of 10-minute duration	The content of the presentation included the following: background to the trial, sample size estimates and monthly recruitment targets, overall recruitment rates across all sites, monthly recruitment rates across all sites, trends in recruitment with a focus on Site A trends.
Discussion of 20-minute duration	Focused on possible reasons for slow recruitment: examples of reasons offered included busyness in the antenatal clinic impacting on information distribution; mindfulness in remembering to distribute; busyness in the labour admission room and the time taken to complete the trial screening and register forms and to obtain consent and randomise participants; and junior staff confidence in recruitment processes and in not performing an ACTG on those women randomised to IA.
Solutions offered	Suggested solutions included a collective and concerted effort to distribute the study information (study information booklets would subsequently be placed in the consulting rooms in addition to being distributed as women checked in); reminders from the consultant obstetricians to their obstetric team to distribute the information; managing the screening process as a usual admission procedure on all women presenting with signs of labour, and senior midwifery staff supporting junior staff in recruiting participants to the trial.

### Description of control

The control sites (Site B and Site C) did not receive a site visit combined with a scheduled meeting. Rather, routine trial activities, such as weekly visits by a member of the research team to collect completed trial documents and visits to facilitate information sessions for new or rotating staff, were maintained. These routine trial activities were ongoing at all three study sites throughout the duration of recruitment in the trial.

### Outcome measures

The primary outcome measures were as follows:

1. Difference in recruitment in each site (A, B and C) from 1 month pre-intervention to 1 month post-intervention
2. Difference in recruitment in each site (A, B and C) from 3 months pre-intervention to 3 months post-intervention

The secondary outcome measures were as follows:

1. Change in adherence to trial procedure (defined as providing eligible women with the trial information booklet during the antenatal period) in each site (A, B and C) from 1 month pre-intervention to 1 month post-intervention.
2. Change in adherence to trial procedure in each site (A, B and C) from 3 months pre-intervention to 3 months post-intervention.

The 1 month and 3 month timeframes were chosen to assess short- and long-term effects of the intervention.

### Data collection

The data of interest (that is, monthly actual recruitment rates and adherence to trial procedure) were extracted from hard copy trial screening and register forms and from the randomisation service database. The randomisation service used in the trial was an automated telephone randomisation service (https://nl.tenalea.net). Randomisations were logged in real time in a central database, which was password protected and accessible to the lead research assistant only. All extracted data were entered into a pre-designed data management table in preparation for analysis.

### Data analysis

Target recruitment rates at each study site had been calculated using a 50% sample population eligibility and assuming that 50% of the eligible population would agree to participate. Due to variations in the sizes of study sites, target recruitment rates varied between sites. Calculations were performed from the time point that the SWAT-1 intervention was implemented (that is, 22 June 2011) to the corresponding monthly dates before and after the intervention. For example, recruitment proportions 1 month pre-intervention were calculated based on recruitment between 22 May and 21 June inclusive; similarly, recruitment proportions 1 month post-intervention were calculated based on recruitment between 22 June and 21 July inclusive. We used the *z*-score test for proportions to determine any statistically significant difference in recruitment within Site A, Site B and Site C between pre-intervention and post-intervention recruitment (H_a_: recruitment pre-intervention < recruitment post-intervention, alpha 0.05, one-tailed). For between-site differences, odds ratios (OR) and 95% confidence intervals (CI) were calculated to compare Site A pre- and post-intervention recruitment and adherence with pre- and post-intervention recruitment and adherence in Site B + Site C combined.

### Ethical approval

Ethical approval was granted for the ADCAR trial by the Faculty of Health Sciences Research Ethics Committee, Trinity College Dublin, the Coombe Women and Infant’s University Hospital Research Ethics Committee, Galway University Hospitals Clinical Research Ethics Committee and by the Healthcare Research Advisory Committee, Health Services Executive, North-East Region. A written signed consent form was obtained from all participants in the ADCAR trial prior to inclusion and randomisation. Ethical approval was not required specifically or in isolation for the site visit because this was a responsive intervention, embedded within the main trial as part of trial procedure to address slow recruitment.

## Results

**Figure 1:**
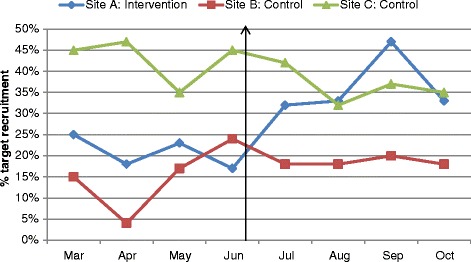
Recruitment rates proportionate to target rates: 2011.

Figure 1 presents an overview of monthly recruitment rates, proportionate to target rates, in all three study sites pre- and post-intervention. The black vertical arrow indicates the time-point of the intervention.

Table 3 presents recruitment rates, proportionate to target recruitment, within all three sites, 1 month and 3 months pre- and post-intervention. The percentage point change and the percentage change in recruitment pre- and post-intervention are provided.

**Table 3:** **Recruitment pre**- **and post**-**intervention**

**Site and time**-**point**	**Pre**-**intervention**	**Post**-**intervention**	**Change from pre**- **to post**-**intervention**		
	**% of target (n/n = actual**/**target)**	**% of target (n/n = actual/target)**	**% point change**	**% change**	***P*** **value**
Site A at 1 month	13% (8/60)	30% (18/60)	+17%	125%	**0.01**
Site A at 3 months	20% (36/180)	34% (61/180)	+14%	69%	**0.002**
Site B at 1 month	27% (32/120)	19% (23/120)	- 8%	39%	0.08
Site B at 3 months	14% (50/360)	18% (65/360)	+4%	30%	0.06
Site C at 1 month	42% (25/60)	43% (26/60)	+1%	4%	0.43
Site C at 3 months	42% (76/180)	38% (60/180)	- 4%	11%	0.23

The analyses demonstrated a 17% and 14% percentage point increase in recruitment in Site A, respectively, at 1 and at 3 months post-intervention. No statistically significant differences (increase or decrease) in recruitment occurred in Site B or in Site C post-intervention at 1 month or at 3 months.

**Figure 2:**
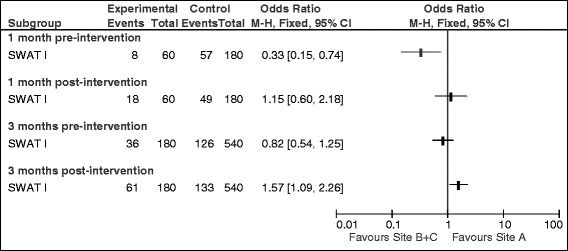
Comparison of actual versus target recruitment in site A versus B + C at 1 and 3 months pre- and post-intervention.

Comparing between-site differences in recruitment, a significantly lower rate of recruitment compared to the target rate was found in Site A (Site A actual recruitment/target recruitment) compared to Site B + C (Sites B plus Site C actual recruitment/target recruitment) at 1 month pre-intervention (13% versus 32%; OR 0.33, 95% CI 0.17 to 0.74). At 1 month post-intervention, this difference was no longer observed (30% versus 27%; OR 1.15, 0.60 to 2.81) (Figure 2). At 3-months pre-intervention, no statistically significant difference in recruitment rates in Site A compared to Site B + C was demonstrated (20% versus 23%; OR 0.82, 95% CI 0.54 to 1.25); however, at 3-months post-intervention, a statistically significant difference was detected in favour of higher recruitment in Site A (34% versus 25%; OR 1.57, 95% CI 1.09 to 2.26) (Figure 2).

Adherence to trial procedure was calculated based on the number of women screened eligible to participate in the trial who had received the study information booklet by the time-point of possible recruitment, divided by the total number of women screened eligible to participate in the study (irrespective of whether they participated or not). These figures were retrieved from the trial screening and register forms completed just prior to the time-point of each woman’s potential recruitment. Comparing within-site adherence, the analyses demonstrated a statistically significant increase in adherence at 1 month and at 3 months post-intervention in Site A (Table 4). There was no significant change (reduction or increase) between pre-intervention and post-intervention adherence rates in Site B and in Site C (Table 4).

**Table 4:** **Adherence to trial procedure pre- and post-intervention**

**Site and time**-**point**	**Pre**-**intervention (number screened eligible who received study information/total screened eligible)**	**Pre-intervention (number screened eligible who received study information/total screened eligible)**	***P*** **value**
Site A at 1 month	58% (15/26)	82% (28/34)	**0.02**
Site A at 3 months	63% (45/72)	87% (90/103)	**<0.0001**
Site B at 1 month	68% (49/72)	72% (54/75)	0.30
Site B at 3 months	66% (107/163)	72% (137/189)	0.08
Site C at 1 month	95% (40/42)	85% (33/39)	0.55
Site C at 3 months	90% (106/118)	88% (84/95)	0.37

**Figure 3:**
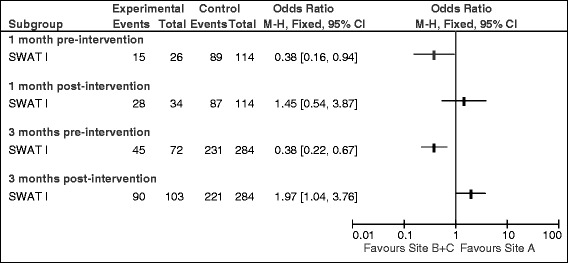
Comparison of adherence between site A and B + C at 1 and 3 months pre- and post-intervention.

Comparing between site differences in adherence rates, a statistically significantly lower adherence rate in Site A compared to Site B + C was found at 1 month pre-intervention (58% versus 78%; OR 0.38, 95% CI 0.16 to 0.94). This difference was not observed at 1 month post-intervention (82% versus 76%; OR 1.45, 95% CI 0.54 to 3.87) (Figure 3). At 3 months pre-intervention, a statistically significantly lower adherence to trial procedure was demonstrated in Site A compared to Site B + C (63% versus 78%; OR 0.36, 95% CI 0.20 to 0.64). Conversely, at 3 months post-intervention, adherence was significantly higher in Site A compared to Site B + C (87% versus 78%; OR 1.97, 95% CI 1.04 to 3.76) (Figure 3).

## Discussion

In this paper, we provide a SWAT-1 evaluation to investigate the effects of a site visit combined with a scheduled meeting, with the sole purpose of discussing trial recruitment, on recruitment rates in a large multi-centre trial. The results provide evidence of benefits from such a site visit for increasing recruitment rates and adherence to trial procedure at 1 and at 3 months after the visit. Other studies have also suggested some benefit for additional communication strategies [5,11]. For example, Monaghan and colleagues [5] investigated the effect of different levels of communication between the trial co-ordination team and participating study sites. Additional communication involved the addition of frequent emails to the usual communications, regular personalised mail-outs of league tables or graphs of performance against other sites and certificates of achievement for recruitment. This was compared to usual communication provided via the regional centre, which involved occasional direct communications in the form of generic newsletters, emails or faxes. At the end of recruitment, there was no significant difference in the median number of participants randomised in each centre in the two groups (37.5 versus 37.0, *P* = 0.68). The median time to half randomisation target was non-significantly lower in the additional communication group compared to the usual group (4.4 months versus 5.8 months, *P* = 0.08), which suggests that an additional communication strategy may be of some, albeit non-statistically significant, incremental benefit in helping sites achieve recruitment targets sooner. In our SWAT-1, the additional communication strategy involved a personalised site visit, suggesting perhaps that face-to-face additional communication might confer more benefit in assisting boost recruitment rates in a trial compared to non-face-to-face strategies.

There are some limitations associated with this implementation of SWAT-1 that need to be acknowledged when considering our results. Firstly, although recruitment rates had been less than expected at all three sites, month-end recruitment at the intervention site had fallen considerably and consistently in the months before the site visit and meeting. Therefore, this site was purposively chosen for the intervention, rather than randomly assigned. This lack of randomisation introduces the potential for bias. Secondly, there is a risk that other confounding factors might have influenced the results. For example, regular weekly visits by a member of the research team were ongoing for all three sites throughout the trial, including the intervention site, Site A. This makes it difficult to disentangle the effect that these routine activities might have had on the findings of our study. For example, one might question as to whether the targeted site visit alone caused the increase in recruitment at 1 and 3 months post-intervention at Site A or whether the targeted site visit increased the impact of the routine visits, such that this combination caused the effect. Thirdly, adherence to trial procedure was evaluated based on information available from completed trial screening and register forms but the accuracy of these findings is dependent on the completeness of reporting of these forms. We cannot be certain that forms were completed on all potentially eligible women; that is, those who did not receive the study information and those who did receive it but did not agree to join the study. Therefore, the results of this analysis should be treated with caution. Furthermore, just prior to implementing the intervention, Site A had taken a downturn in recruitment with a subsequent upturn, while Site B and Site C had taken an upturn with a subsequent downturn (Figure 1), consistent with regression to the mean. However, it is worth noting that, for Site A, recruitment is maintained long-term at a considerably higher rate than pre-intervention, which is promising. Finally, this SWAT-1 evaluation uses a retrospective design in which the occurrence of the intervention was combined with recruitment rates sometime after the site visit and meeting. Ideally, to enhance rigour, SWAT-1 should be prospectively implemented and include random allocation of study sites to intervention and control. However, randomisation is not always possible and a before and after evaluation is worthwhile. If several before and after studies all show a similar effect, it might be reasonable to consider that this is evidence of benefit.

Recognising these limitations, our study does provide some evidence for the effectiveness of a site visit combined with a scheduled meeting on recruitment rates and adherence to trial procedure in a multi-centre randomised trial. More importantly, perhaps, our study demonstrates the potential value of using SWAT-1 to evaluate, pragmatically, what works when attempting to tackle the problem of poor recruitment to a trial. Based on our results, other researchers might also be encouraged to implement SWAT-1, or their own version of it, if they are facing similar problems of slow or poor recruitment in their trials. As the findings from further SWAT-1 s become available, the results could be combined in cumulative meta-analyses [12], which will provide increased power to estimate the effects of a site visit by the lead researcher or principal investigator on recruitment in multi-centre trials, and to compare and contrast the findings in different settings.

## Conclusions

This paper provides the first reported example of a study in the SWAT programme, using the SWAT-1 design. It provides some evidence that a targeted site visit combined with a purposeful meeting increases recruitment rates and adherence to trial procedure in a clinical trial. We hope that this example of SWAT-1 might encourage other researchers to consider conducting a similar study to improve recruitment in their trials, allowing the findings of future SWAT-1 s to be compared, contrasted and combined so that higher level evidence on the effect of a site visit by the lead researcher or principal investigator can be obtained.

## Abbreviations

ACTG: admission cardiotography
ADCAR: admission cardiotocography versus intermittent auscultation of the fetal heart rate
CI: confidence interval
FHR: fetal heart rate
IA: intermittent auscultation
OR: odds ratio
SWAT: Study Within A Trial
